# Ferristatin II Efficiently Inhibits SARS-CoV-2 Replication in Vero Cells

**DOI:** 10.3390/v14020317

**Published:** 2022-02-03

**Authors:** Alexey Sokolov, Irina Isakova-Sivak, Natalia Grudinina, Daria Mezhenskaya, Elena Litasova, Valeria Kostevich, Ekaterina Stepanova, Alexandra Rak, Ivan Sychev, Olga Kirik, Larisa Rudenko

**Affiliations:** Institute of Experimental Medicine, 197376 Saint Petersburg, Russia; biochemsokolov@gmail.com (A.S.); strangecatnap@gmail.com (N.G.); dasmez@iemspb.ru (D.M.); llitasova@mail.ru (E.L.); Hfa-2005@yandex.ru (V.K.); fedorova.iem@gmail.com (E.S.); alexandrak.bio@gmail.com (A.R.); atheneem@yandex.ru (I.S.); olga_kirik@mail.ru (O.K.); vaccine@mail.ru (L.R.)

**Keywords:** SARS-CoV-2, transferrin receptor 1, ferristatin II, receptor-binding domain (RBD), Vero cells

## Abstract

Severe acute respiratory syndrome coronavirus 2 (SARS-CoV-2) continues to have a significant impact on global public health. Multiple mechanisms for SARS-CoV-2 cell entry have been described; however, the role of transferrin receptor 1 (TfR1) in SARS-CoV-2 infection has received little attention. We used ferristatin II to induce the degradation of TfR1 on the surface of Vero cells and to study the consequences of such treatment on the viability of the cells and the replication of SARS-CoV-2. We demonstrated that ferristatin II is non-toxic for Vero cells in concentrations up to 400 µM. According to confocal microscopy data, the distribution of the labeled transferrin and receptor-binding domain (RBD) of Spike protein is significantly affected by the 18h pretreatment with 100 µM ferristatin II in culture medium. The uptake of RBD protein is nearly fully inhibited by ferristatin II treatment, although this protein remains bound on the cell surface. The findings were well confirmed by the significant inhibition of the SARS-CoV-2 infection of Vero cells by ferristatin II with IC_50_ values of 27 µM (for Wuhan D614G virus) and 40 µM (for Delta virus). A significant reduction in the infectious titer of the Omicron SARS-CoV-2 variant was noted at a ferristatin II concentration as low as 6.25 µM. We hypothesize that ferristatin II blocks the TfR1-mediated SARS-CoV-2 host cell entry; however, further studies are needed to elucidate the full mechanisms of this virus inhibition, including the effect of ferristatin II on other SARS-CoV-2 receptors, such as ACE2, Neuropilin-1 and CD147. The inhibition of viral entry by targeting the receptor on the host cells, rather than the viral mutation-prone protein, is a promising COVID-19 therapeutic strategy.

## 1. Introduction

The coronavirus disease 2019 (COVID-19) pandemic caused by severe acute respiratory syndrome coronavirus 2 (SARS-CoV-2) continues to pose a significant socioeconomic burden to the world’s population. Angiotensin-converting enzyme 2 (ACE2) is considered the main host cell receptor that SARS-CoV-2 utilizes to enter cells [1,2,3,4]; however, several alternative receptors and co-receptors capable of facilitating SARS-CoV-2 cell entry and infectivity have been identified, such as Neuropilin-1, CD147, tyrosine-protein kinase receptor UFO (AXL) and others [5,6,7,8,9,10]. Furthermore, two proteolytic cleavage steps are required for effective SARS-CoV-2 entry into cells after the engagement of a host cell receptor by a viral spike (S) protein. The first step involves the cleavage of the S1–S2 boundary by furin protease within an infected cell, while the S2′ site cleavage is dependent on target cell proteases, such as TMPRSS2 and cathepsin L, which activate S proteins at the plasma membrane and in the endolysosome, respectively (reviewed in [11]). 

Aside fromthe well-studied SARS-CoV-2 host cell entry targets, there is evidence that transferrin receptor 1 (TfR1) can be an alternative co-receptor for SARS-CoV-2 entry [12]. TfR1, a 95 kDahomodimeric type II membrane glycoprotein, is abundantly expressed on the plasma membrane of most human cells and mediates the entry of transferrin (Tf) into cells for the delivery of iron [13]. Indeed, there is a wide range of human and zoonotic viruses that can invade host cells via the interaction with TfR1 and other factors in the iron uptake pathway, including New World arenaviruses, human immunodeficiency virus, parvoviruses, hepatitis C virus, alphaviruses and others [14]. Therefore, targeting the TfR1-mediated viral entry pathway represents a promising therapeutic strategy for SARS-CoV-2 infection. Early screening studies identified two small-size compounds (NCI11079 and NCI306711) that had a significant inhibitory effect on Tf-mediated iron uptake by HeLa cells. Compound NCI306711 targets TfR1 by inducting its internalization and degradation and was named “ferristatin” due to its unusual property of inhibiting iron transport through the cholesterol-dependent pathway [15]. Another small molecule, ferristatin II (NSC8679), acts in a manner similar to ferristatin and degrades TfR1 through a nystatin-sensitive lipid raft pathway [16]. In light of these data, we attempted to assess the ability of ferristatin II to inhibit SARS-CoV-2 replication in Vero-CCL81 cells and to explore the potential mechanisms of this inhibition.

## 2. Materials and Methods

### 2.1. Viruses, Proteins and Chemical Compounds

The SARS-CoV-2 isolates hCoV-19/St_Petersburg-3524S/2020 (Wuhan D614G lineage), hCoV-19/Russia/SPE-RII-32759V/2021 (Delta lineage) and hCoV-19/Russia/SPE-RII-6086V/2021 (Omicron lineage) were obtained from Smorodintsev Research Institute of Influenza (Saint Petersburg, Russia). All work with infectious viruses was performed in biosafety level 3 (BSL-3) laboratory.

A recombinant protein corresponding to the receptor-binding domain (RBD) of SARS-CoV-2 S protein was expressed in eucaryoticcells by BIOCAD JSC (St.Petersburg, Russia). 

Iron-saturated human serum TF (Fe_2_-TF) was isolated from human blood serum by ion-exchange chromatography on DEAE-Toyopearl, UNOsphere S, and gel filtration on Sephadex G-75 Superfine, as previously described [17].

**Figure 1:**
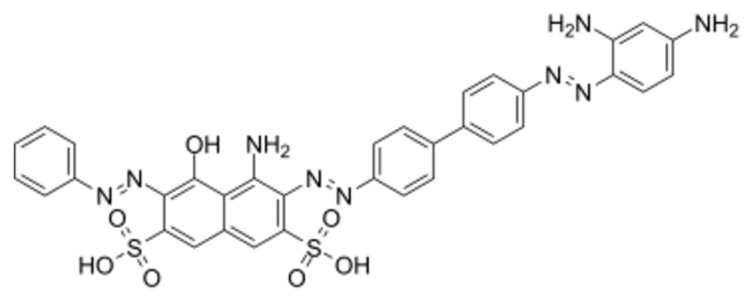
The structure of ferristatin II molecule (adopted from https://www.chemsrc.com/en/cas/22244-14-0_1559138.html, accessed on 31 December 2021).

Ferristatin II (Figure 1) was purchased from Sigma (C-1144, St. Louis, MO, USA). The compound was diluted in 150 mM NaCl, 10 mM Na-phosphate buffer, pH 7.4 (PBS) to the final concentration 4 mM, and the solution was filtered through a sterile membrane with pore size 0.22 µm (Sartorius, Germany).

### 2.2. SARS-CoV-2 Virus Propagation and the Assessment of Ferristatin II Virus Inhibition Capacity

Viruses were propagated on Vero CCL81 cells using DMEM supplemented with 1× antibiotic-antimycotic, 10 mM HEPES and 2% FBS (all from Capricorn, Ebsdorfergrund, Germany), (DMEM/2%FBS). Infectious titers were determined by 50% Tissue Culture Infection Dose (TCID_50_) assay, as described in [18] and expressed in lgTCID_50_/mL.

For evaluation of ferristatin virus neutralization capacity, we used a modified microneutralization assay. The day before the experiments, Vero cells were seeded in 96-well culture plates (3 × 10^4^ cells per well) in DMEM supplemented with 1× antibiotic-antimycotic and 10% FBS. The next day, the medium was removed, and the cells were infected with 100 TCID_50_ of SARS-CoV-2 prepared on DMEM/2%FBS for 2 h at 37 °C, 5% CO_2_. Several wells remained uninfected as a negative control. Then, the inoculum was removed, and cells were covered with 150 µL of two-fold ferristatin II dilutions prepared on DMEM/2%FBS, whereas negative and positive control wells were covered with media only. Each ferristatin II dilution was assessed in triplicates. The plates were incubated for 48 h (for Wuhan G614D virus) or 72 h (for Delta and Omicron viruses) at 37 °C, 5% CO_2_, until a clear cytopathic effect was seen in positive control wells. The supernatants were carefully collected into 0.2 mL tubes for viral titer quantification and the cells were fixed with 10% formaldehyde at 4 °C for 24 h. The fixed plates were transferred to the BSL-2 laboratory for a cell-based ELISA procedure. This included washing with PBST (PBS with 0.05% of Tween 20), cell permeabilization with 0.1% Triton X-100 at room temperature for 15 min, blocking with 3% skimmed milk at 37 °C for 1 h, and intensive washing with PBST. Then, purified polyclonal rabbit anti-RBD antibody (BIOCAD JSC, Saint Petersburg, Russia) was added at a concentration 1.5 µg/mL for 1 h, followed by washing with PBST and the addition of secondary anti-rabbit anti-IgG HRP-conjugated antibody (BioRad, Hercules, CA, USA) for 1 h at 37 °C. The plates were developed using 1-Step Ultra TMB-ELISA Substrate Solution (Thermo Fisher Scientific, Waltham, MA, USA). The reaction was stopped with 1M H_2_SO_4_ solution, and the results were read at 450 nm using xMark Microplate Spectrophotometer (BioRad, Hercules, CA, USA). To calculate the IC_50_ titer, four-parametrical non-linear regression analysis was used, based on the OD_450_ values of virus-negative and virus-positive cells, as described in [18].

SARS-CoV-2 titers in the cell supernatants were calculated by quantitative real-time RT-PCR. The RNA was purified from 100 µL of cell culture medium, as well as from SARS-CoV-2 with known titers for the generation of the assay standard curves. RNA samples were purified with RNA extraction kit (Biolabmix, Novosibirsk, Russia) according to manufacturer’s protocol. The RT-PCR reaction was performed in duplicates with forward primer 5′-CAATGGTTTAACAGGCACAGG-3′ and reverse primer 5′-CTCAAGTGTCTGTGGATCACG-3′ [19] using One-Tube RT-PCR SYBR kit (Evrogen, Moscow, Russia) on Quantstudio 1 Real-Time PCR System (Thermofisher Scientific, Waltham, MA, USA), with addition of 50x Rox as a reference dye. The threshold cycle (Ct) was calculated for each sample with QuantStudio Design and Analysis Software v1.5.1, and the standard curve was generated on each reaction plate. The titer of the virus in each well was calculated using Ct values normalized to standard curves calculated on the base of standard virus titration and expressed in lgTCID_50_/mL.

To determine whether ferristatin II can inhibit initial steps of viral attachment to the cells, 100 TCID_50_ of SARS-CoV-2 were mixed with ferristatin II dilutions, and after 1h incubation at 37 °C, this mixture was added to Vero cells, followed by an additional 1h incubation at 37 °C. After viral absorption, the inoculum was removed, and the cells were covered with 150 µL of fresh DMEM/2%FBS media. After 48 h of incubation at 37 °C, 5% CO_2_ and visible CPE in positive control cells, plates were fixed and subjected to cell-based ELISA as described above.

### 2.3. Assessment of Cytotoxic Effect of Ferristatin II

Cytotoxicity of ferristatin II for Vero cells was assessed by the 3-(4,5-Dimethylthiazol-2-yl)-2,5-diphenyltetrazolium bromide (MTT) reduction assay [20]. Two-fold dilutions of ferristatin II were prepared in DMEM/2%FBS medium starting from 400 µM concentration and added to the wells of 96-well plates with Vero cell monolayers. After 48h incubation, the media were removed and the cells were gently washed with warm PBS. The 0.5 mg/mL MTT reagent was added to each well in a volume of 100 µL, and the plate was further incubated at 37 °C for 2 h. Then, the reagent was removed and the cells were washed with PBS and air-dried, followed by the addition of 100 µL of 96% ethanol to dissolve formazan crystals. The color intensity was recorded using a spectrophotometer at a wavelength of 535 nm. Wells of the plate not seeded with Vero cells were used as negative controls. The 50% inhibition of cell viability (CC_50_) was calculated by four-parametrical non-linear regression analysis using GraphPad Prizm software.

### 2.4. Labeling of Human Serum Transferrin and Recombinant RBD Protein

The labeling reaction was carried out at +4 °C for 12 h by adding 5 mM solution of NHS-ester 5-TAMRA (Lumiprobe, #47120) in dimethylsulfoxide to 30 µM Fe_2_-TF or RBD solutions in 200 mM NaHCO_3_, keeping the ratio 5-TAMRA:protein = 8:1 (mol/mol). After removal of excess dye, the degree of labeling was estimated by the ratio of A_541_ and A_280_, which was 3.5 and 3.2 mol TAMRA per mol Fe_2_-TF and RBD, respectively.

### 2.5. Study of Ferristatin II Effect of TF and RBD Uptake by Vero Cells

Vero cells were cultured for 18 h in DMEM/2%FBS without ferristatin II and with the addition of 100 µM ferristatin II with coverslips for microscopy previously placed on the bottom of the culture plates. Then, the medium was replaced for 1.5 h with medium without FBS containing 1.2 µM Fe_2_-TF-TAMRA (0.25 mg/mL) or 1.2 µM RBD-TAMRA (0.1 mg/mL). After incubation, the cells were gently washed twice with PBS, fixed with 4% paraformaldehyde (*w*/*v*) in PBS for 10 min, washed twice with PBS for 30 min. Then, nuclei of the cells were stained with 360 nM DAPI for 5 min, washed with PBS and coverslips, and mounted on the slides using a polymerizing resin for fluorescence microscopy (DAKO, Glostrup, Denmark). Images were acquired using an Axio Obserever.Z1/7 confocal laser scanning microscope (Carl Zeiss, Jena, Germany), with a Plan-Apochromat 63×/1.40 Oil DIC M27 objective. A 561 nm laser was used to excite TAMRA fluorescence, a 570–700 nm filter was used to record fluorescence, a 405 nm laser was used to excite DAPI, and a 400–570 nm filter was used to record fluorescence. Images were acquired and processed using the software provided with the microscope.

## 3. Results

**Figure 2:**
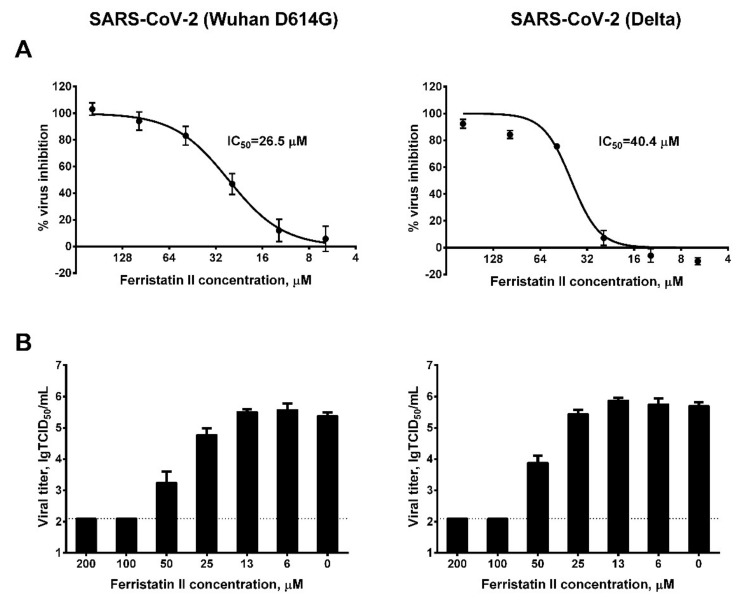
Inhibition of SARS-CoV-2 replication in Vero cells by ferristatin II. (**A**) Assessment of the levels of virus neutralization by the detection of RBD antigen in the cells by cell-based ELISA. (**B**) Assessment of viral titers in cell supernatants by quantitative real-time RT-PCR. The dotted line represents the limit of viral RNA detection. IC_50_: 50% inhibiting concentration. Data are presented as the mean ± SD (*n* = 3).

**Figure 3:**
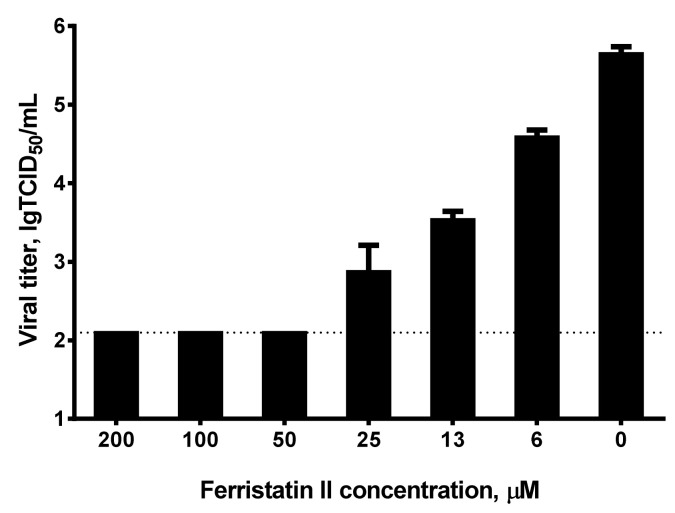
Inhibition of SARS-CoV-2 Omicron variant replication in Vero cells by ferristatin II. Infectious viral titers in cell supernatants were measured by quantitative real-time RT-PCR. The dotted line represents the limit of viral RNA detection. Data are presented as the mean ± SD (*n* = 3).

We used two antigenically distant SARS-CoV-2 strains to assess the ability of ferristatin II to inhibit viral replication in Vero cells. At the first stage, we assessed viral inhibition in the constant presence of the compound in culture medium. The detection of viral antigen in cell-base ELISA found that the IC_50_ values for ferristatin II were 26.5 and 40.4 µM for the Wuhan D614G and Delta viruses, respectively (Figure 2A). The quantification of the virus titers in culture media confirmed that the release of the virus from the infected cells into the culture medium was inhibited by ferristatin II, starting from 25 µM concentration, and IC_50_ values calculated from the reduction in TCID_50_ titers were 24.0 and 25.2 µM for the Wuhan D614G and Delta viruses, respectively. Moreover, when the compound was added at a concentration of 100 µM, no viral replication occurred in Vero cells (Figure 2B). Due to the significant differences in the RBD proteins of the Wuhan D614G and the Omicron SARS-CoV-2 variants, the latter could not be detected in cell-based ELISA using the available anti-RBD antibody, as it was raised to the RBD of original SARS-CoV-2 strain. Nevertheless, the reduction in infectious viral titers in the presence of ferristatin II was even more pronounced in the Omicron variantcompared to the other two SARS-CoV-2 strains: a tenfold decrease in the viral titer was noted at a ferristatin II concentration as low as 6.25 µM, with no virus replication occurring at concentrations of 50 µM and above (Figure 3). 

It is important to note that this inhibition of SARS-CoV-2 occurred only when ferristatin II was constantly present in the culture medium, whereas this effect was not observed when the cells were pretreated with the compound or when a virus/ferristatin II mixture was used in the adsorption step followed by incubation without the substance (data not shown).

**Figure 4:**
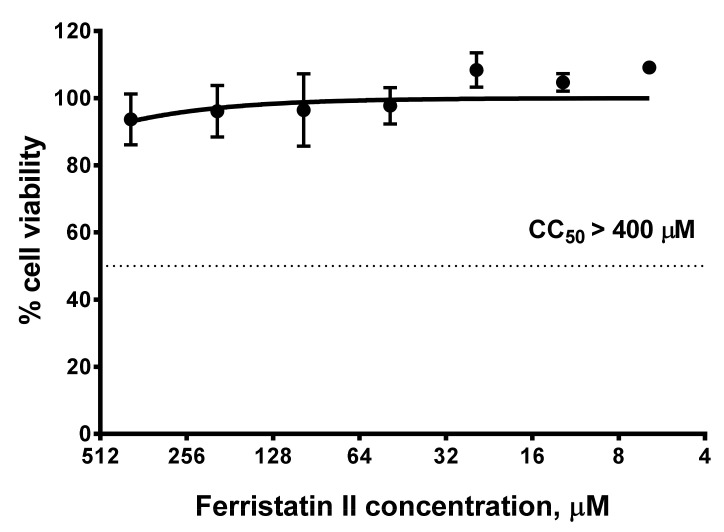
Cytotoxicity of ferristatin II for Vero cells. The cells were incubated for 2 days in the presence of indicated concentrations of ferristatin II, followed by the assessment of cell viability using MTT assay. Data are presented as the mean ± SD (*n* = 3).

We further assessed the cytotoxic effect of ferristatin II on Vero-CCL81 cells. The presence of this compound in the culture medium at a concentration up to 400 µM had no negative effect on the viability of the cells, suggesting that the inhibition of SARS-CoV-2 replication was not due to the cell’s metabolic dysfunctions (Figure 4). Therefore, the selectivity indices (determined as a ration of CC_50_ to IC_50_ values) for ferristatin II were >10, >15 and >64 for Wuhan D614G, Delta and Omicron SARS-CoV-2 strains, respectively.

**Figure 5:**
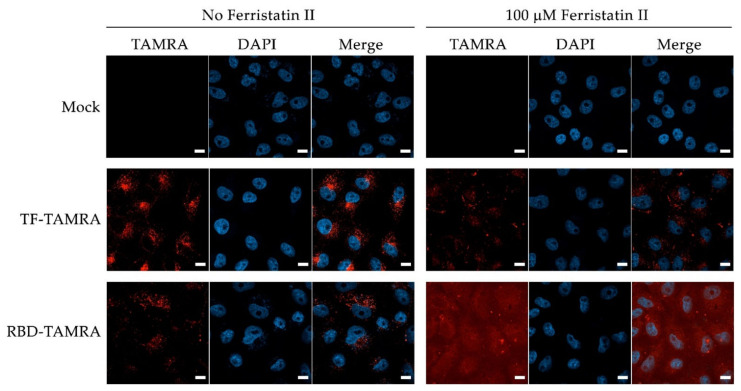
Confocal images showing Fe_2_-TF and RBD binding and uptake to Vero cells without treatment (Mock) and with treatmentfor 18 h by 100 µM Ferristatin II. TAMRA-labeled Fe_2_-TF and RBD (1.2 µM) were added to cells for 1.5 h. Nuclei were stained by DAPI. Scale bar: 10 µm. The micrographs shown are representative of three separate experiments, fixation and details of microscopy, are described in Materials and Methods.

To explore the possible mechanisms of SARS-CoV-2 inhibition by ferristatin II, we studied the effect of the substance on viral entry by a surrogate RBD uptake assay. Thus, we determined how 100 µM non-toxic concentration of ferristatin II can affect the uptake of tetramethylrhodamine-labeled (TAMRA) RBD by Vero cells. TfR1, a target for ferristatin II action, is known to mediate Tf entry; therefore, the TAMRA-labeled TF was used as a positive control. Culturing Vero cells in the presence of 100 µM ferristatin II for 18 h without adding labeled proteins had no significant effect on the morphology of the cell nuclei (Figure 5, top panel (Mock)), indirectly indicating the absence of cell necrosis and apoptosis. The incubation of cells with TF-TAMRA without ferristatin II treatment resulted in accumulations of labeled proteins inside the cells, without affecting the DAPI-stained nuclear region (left middle triad of images (TF-TAMRA) in Figure 5). The treatment of cells with 100 µM ferristatin II resulted in a significant reduction in the inclusion of labeled TF (right middle triad of images (TF-TAMRA) in Figure 5). The incubation of cells with RBD-TAMRA without ferristatin II treatment, as in the case of labeled TF, resulted in the accumulation of labeled proteins in the cell cytoplasm, without affecting the nuclear region (bottom left triad (RBD-TAMRA) in Figure 5). When the cells were treated with 100 µM ferristatin II, a redistribution of the TAMRA signal occurred. Instead of incorporating the labeled RBD into the cells, the TAMRA-signal was distributed evenly over the cell surface, with only a small portion of the label forming clusters partially overlapping the DAPI-staining of the nuclei, indicating a surface localization of the TAMRA label (bottom right triad (RBD-TAMRA) in Figure 5). The results shown in Figure 5 indicate that the treatment of Vero cells with 100 µM ferristatin II affects the incorporation of the labeled RBD into the cells. Considering that no data are currently available on the effect of ferristatin II on the ACE2 receptor, it cannot be excluded that the observed effect is due to the binding of labeled RBD to the cell surface, but the absence of TfR1 on the cells prevents the incorporation of RBD in the cells treated with ferristatin II. 

## 4. Discussion

SARS-CoV-2 utilizes multiple mechanisms to invade host cells and cause successful viral infection, and a wide range of host cell entry inhibitors are currently being assessed as COVID-19 therapeutic strategies [21]. In the current study, we report preliminary results of inhibiting SARS-CoV-2 in vitro replication by the treatment of the target cells by ferristatin II, a small-size molecule that can selectively degrade transferrin receptor 1. Iron-saturated transferrin (Fe_2_-Tf or holo-Tf) possesses a high affinity to its receptors, which is expressed on the surfaces of most human cells and is internalized in complex with holo-Tf by receptor-mediated endocytosis. The formation of the complexes between iron and Tf, as well as between holo-Tf and TfR, are pH-dependent and provide the release of iron from Tf and Tf from TfR in the acidic environment of endosome. The recycling of endosome results in TfR returns to the plasma membrane and releases apo-form of Tf for a further round of irondelivery to the cell. The uptake of iron by mammalian cells is an important processfor regulating the growth of cancer cells that lack pharmacological agents with known mechanisms of action. A screening of 2000 small-size compounds from the National Cancer Institute’s Diversity Set library identified 10 inhibitors of iron transport with IC_50_ values ranging from 5 to 30 µM [22]. Ferristatin II has been used as a blocker of TfR1 in studies of cell infection via TfR1 with the hepatitis C virus [23,24], transmissible gastroenteritis virus [25], and reovirus [26] (Table 1). It should be noted that experiments on TfR1 degradation with ferristatin II are almost always carried out in the permanent presence of the substance in the medium. 

In a similar assay setup, we found that ferristatin II was virtually nontoxic for Vero cells during 48h exposure to a concentration of 400 μM. This concentration is sufficient for a complete degradation of TfR1 on the surface of cultured cells [16]. On the one hand, this effect was confirmed by the reduced uptake of labeled Fe_2_-Tf by Vero cells pretreated with 100 µM of ferristatin II for 18 h. The non-complete blocking of labeled Tf uptake in this case can be explained by the absence of ferristatin II at the time of the addition of labeled proteins. On the other hand, the treatment of Vero cell by ferristatin II dramatically changed the uptake of the RBD of Spike proteins. In this preliminary study, we didnot assess the level of expression of other SARS-CoV-2 receptors under ferristatin II treatment, and did not induce the knock-out of these known receptors to prove the independent role of TfR in mediating SARS-CoV-2 cell entry, which is an area of our further research. Nevertheless, a relatively uniform binding of RBD to the surface of the cells treated with ferristatin II may indicate that some of the SARS-CoV-2 receptors on Vero cells remain functional and bind the virus, but in the absence of TfR1, the virus cannot enter the cell. This receptor is most likely ACE2, since Prabhakara et al. demonstrated an increase in the uptake of labeled RBD along with labeled transferrin by cells overexpressing ACE2, suggesting that ACE2 promotes RBD uptake through clathrin-mediated endocytosis, which is exploitedby TfR1 [28]. Furthermore, there is evidence that SARS-CoV-2 can infect ACE2-negative cells using alternative entry pathways [29]; therefore, a deeper analysis of ferristatin II action on various cell types permissive for SARS-CoV-2 infection will shed light on the role of TfR1 in the virus life cycle.

To understand how promising this substance is for the future therapy of SARS-CoV-2 infection, it is important to know how this compound is metabolized in vivo. Rats treated with ferristatin II had lower levels of TfR1 in liver, and reduced serum iron and Tf saturation without changes in liver non-heme iron [16]. Furthermore, ferristatin II induced the expression of hepcidin [16,30]. In another study, gene expression was assessed after the administration of ferristatin II (in corn oil) into the stomachs of Wistar rats at a dose 146 mg/kg/day for 7 days. Among the genes of iron-regulating proteins, a decreased expression of HFE and increased expression of CP, GDF15 and HAMP after ferristatin II administration was observed [31]. Of note, the effect of ferristatin II on the p53-dependent signaling pathway was most likelythe barrier limiting its use only as a TfR1 inhibitor on cell cultures [31].

Ferristatin II is also known as direct black 38, Chlorazol black E, C.I. 30235, or CAS 1937-37-7 and is commonly used as a dye for staining granulocytes and onychomycosis [32,33]. Importantly, benzidine-metabolites of direct black 38 that possess a potential risk of bladder cancer were observed in urine samples of workers in a small-scale unit that manufactured this dye [34]. Although ferristatin II cannot be directly used in humans for this reason, there is argument that the strategy of inhibiting SARS-CoV-2 entry into the cell by blocking TfR1 is promising, since this approach targets the receptor on the host cells rather than the viral mutation-prone protein. Additionally, further studies are warranted to identify chemical compounds that act like the ferristatin II molecule, but without the formation of metabolites with potential health risks.

## Figures and Tables

**Table 1:** Examples of ferristatin II effects on the replication of human and animal viruses.

Virus	Ferristatin Dose, µM	Time of Treatment	Effect	Reference
Hepatitis C virus (HCV)	25–50	20 h	Significant reduction in HCV cell-to-cell spread observed in ferristatin-treated Huh7 cells	[23]
Transmissible gastroenteritis virus (TGEV)	50	24 h	3-fold reduction in viral TGEV-N protein level in IPEC-J2 cells	[25]
Porcine epidemic diarrhea virus (PEDV)	50	3h	3-fold reduction in PEDV titer and RNA level in IPEC-J2 and Vero cells	[27]
Glass carp reovirus (GCRV)	100	24 h	(2–3)-fold increase in expression of interferons (IFN-1 and IFN-3) in GCRV-infected CIK cells	[26]

## Data Availability

The data presented in this study are available on request from the corresponding author.
